# Abnormal dendritic maturation of developing cortical neurons exposed to corticotropin releasing hormone (CRH): Insights into effects of prenatal adversity?

**DOI:** 10.1371/journal.pone.0180311

**Published:** 2017-06-28

**Authors:** Megan M. Curran, Curt A. Sandman, Elysia Poggi Davis, Laura M. Glynn, Tallie Z. Baram

**Affiliations:** 1 Department of Anatomy & Neurobiology, University of California Irvine, Irvine, California, United States of America; 2 Department of Psychiatry and Human Behavior, University of California Irvine, Irvine, California, United States of America; 3 Department of Psychology, University of Denver, Denver, Colorado, United States of America; 4 Department of Psychology, Chapman University, Orange, CA, United States of America; 5 Department of Pediatrics, University of California Irvine, Irvine, California, United States of America; 6 Department of Neurology, University of California Irvine, Irvine, California, United States of America; Radboud University Medical Centre, NETHERLANDS

## Abstract

Corticotropin releasing hormone (CRH) produced by the hypothalamus initiates the hypothalamic-pituitary-adrenal (HPA) axis, which regulates the body’s stress response. CRH levels typically are undetectable in human plasma, but during pregnancy the primate placenta synthesizes and releases large amounts of CRH into both maternal and fetal circulations. Notably, placental CRH synthesis increases in response to maternal stress signals. There is evidence that human fetal exposure to high concentrations of placental CRH is associated with behavioral consequences during infancy and into childhood, however the direct effects on of the peptide on the human brain are unknown. In this study, we used a rodent model to test the plausibility that CRH has direct effects on the developing cortex. Because chronic exposure to CRH reduces dendritic branching in hippocampal neurons, we tested the hypothesis that exposure to CRH would provoke impoverishment of dendritic trees in cortical neurons. This might be reflected in humans as cortical thinning. We grew developing cortical neurons in primary cultures in the presence of graded concentrations of CRH. We then employed Sholl analyses to measure dendritic branching and total dendritic length of treated cells. A seven-day exposure to increasing levels of CRH led to a significant, dose-dependent impoverishment of the branching of pyramidal-like cortical neurons. These results are consistent with the hypothesis that, rather than merely being a marker of prenatal stress, CRH directly decreases dendritic branching. Because dendrites comprise a large portion of cortical volume these findings might underlie reduced cortical thickness and could contribute to the behavioral consequences observed in children exposed to high levels of CRH in utero.

## Introduction

Corticotropin-releasing hormone (CRH), a hypothalamic, 41-amino acid neuropeptide, has a major role in regulating pituitary–adrenal function and biological responses to stress [1–6]. The expression of CRH in stress-sensitive hypothalamic neurons commences during fetal life [7–9], and the peptide contributes to the regulation of the response to stress throughout life. In addition to expression and function within the hypothalamus, CRH is expressed in select brain regions [10,11], and CRH receptors are distributed throughout the brain [12,13]. During stress, the peptide is released locally within the amygdala [14], hippocampus [15], and cortex [16], and contributes to the diverse effects of stress on brain functions, including memory and anxiety. Thus, during acute stress, CRH enhances memory through actions in the amygdala [14] and hippocampus [17]. However, exposure to high levels of CRH, or a chronic exposure to lower levels may have adverse effects throughout life, because the peptide reduces the number of neuronal synapses by disrupting the structure of dendritic spines [18,19]. During aging, CRH may also contribute to the pathology associated with dementia, including Alzheimer’s Disease [20,21]. Developing neurons are particularly sensitive to CRH. Indeed, exposure to nanomolar levels of the peptide results in neuronal hyper-excitability [22], increased risk for seizures, and even neuronal death [23,24].

There is experimental information about the role of brain-derived CRH during development; however, there is little known about the potential role of placental-derived, maternal CRH on brain maturation in the developing fetus. Rodent placenta does not seem to synthesize CRH. In contrast, during the course of human pregnancy the placenta expresses CRH as early as the seventh week of gestation [25]. This results in an exponential (20- to 40-fold) increase in CRH levels in maternal and fetal circulation over the course of human gestation. Placental CRH (pCRH) is identical to hypothalamic CRH, and it is believed that CRH contributes to organization of the fetal nervous system, regulates fetal maturation [26]^,^ and influences the timing of birth [27,28]. In the human, maternal signals of stress (e.g. increased cortisol) is associated with augmented synthesis of pCRH that is released into both maternal and fetal compartments.

**Fig 1:**
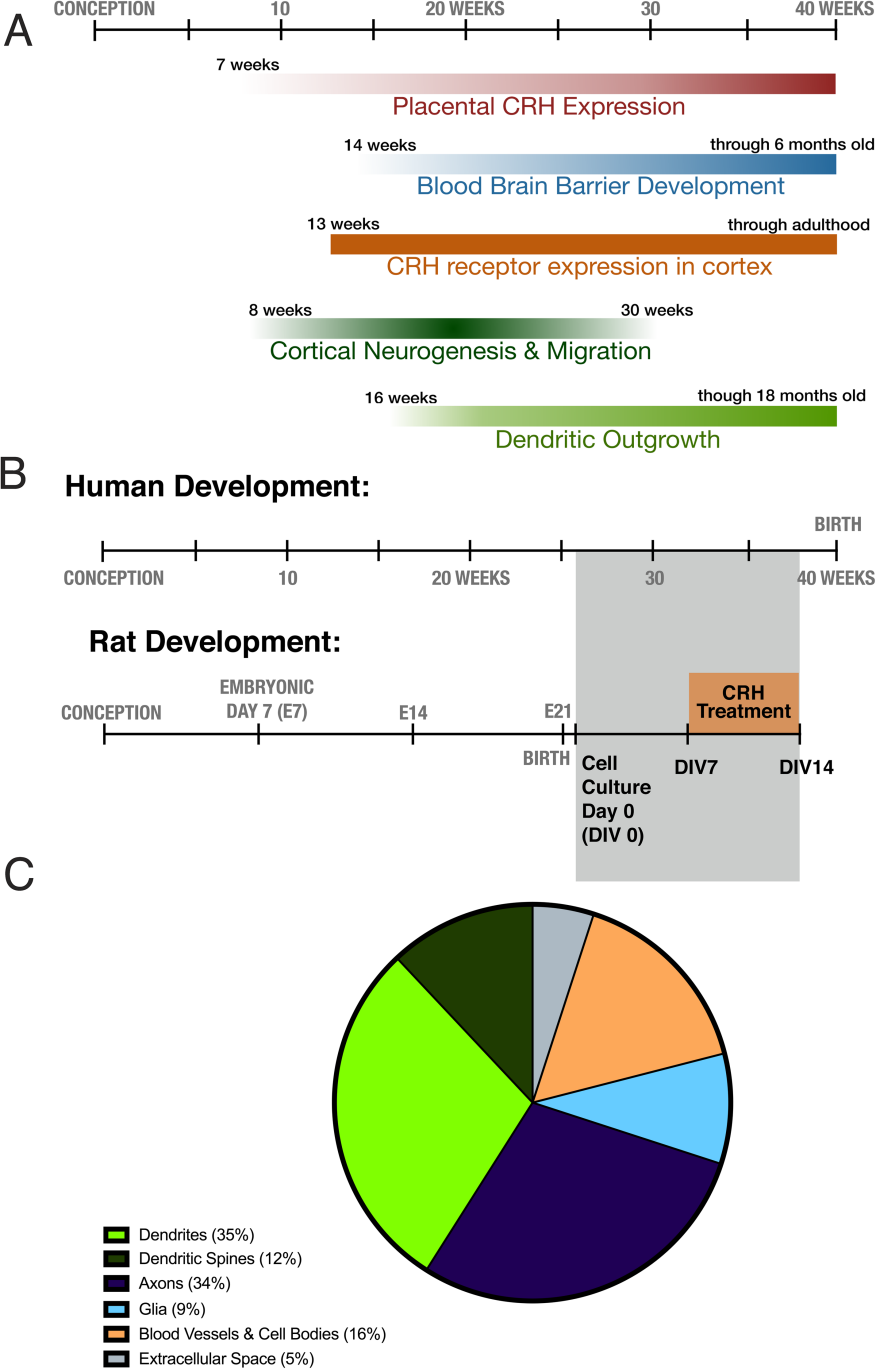
The relative timing and cortical volume impacts of CRH on dendritic growth in the fetus. A) The relative timing of placental CRH expression, blood brain barrier development, cortical CRH expression, and cortical neurogenesis, migration and dendritic outgrowth in human gestation [25,32–34]. B) Relative human and rat cortical neuronal development. The relative timing of the cell culture experiment and treatment with CRH is indicated by the grey and orange boxes, respectively. C) Contribution of different cell types and structures to cortical volume. Based on data from Braitenberg & Schüz (1998).

An association has been found between fetal exposure to elevated level of CRH and childhood outcomes. For example, increased pCRH exposure has been correlated with internalizing symptoms in children [29,30], but how CRH promotes these outcomes is unclear. The peptide might mainly function via influencing glucocorticoids and other mediators; alternatively, CRH may directly influence the development and maturation of brain cells, including neurons. This question is important: in addition to mechanistic understanding of human brain development, it may open therapeutic opportunities. Here, we query if, in addition to being a marker of prenatal stress, CRH exposure during periods of cortical neuronal development and growth might directly stunt the dendritic trees of rodent cortical neurons (Fig 1A). Using an *in vitro* model, we find a deleterious, dose-dependent effect of CRH on the development of cortical neurons, at a developmental period approximating human developmental stages when pCRH levels are maximal (Fig 1B). CRH led to poor development of dendritic trees, which constitute ~45% of cortical volume [31] (Fig 1C) and are essential for anchoring excitatory synapses, the basis of normal neuronal function. Thus, CRH-mediated mal-development of vital cortical structures may contribute to the mechanisms by which prenatal adversity may influence human neuropsychiatric outcome.

## Methods

All animal experiments conformed to National Institutes of Health guidelines and were approved by the Institutional Animal Care and Use Committee of the University of California-Irvine.

The detailed protocol has been listed for independent citation at protocols.io [35].

### Primary cortical neuron cultures

Timed-pregnant Sprague-Dawley rat dams gave birth in the University of California-Irvine vivarium. Three independent experiments were conducted: Cortical neuron cultures were prepared within 36 hours after birth (P0-P1) from pups of either sex as previously described [36,37]. Briefly, motor cortices were dissected and incubated in dissection solution (137 mM NaCl, 5.4 mM KCl, 0.17 mM Na_2_PO_4_, 0.22 mM KH_2_PO_4_, 33.3 mM D-glucose, and 43.8 mM sucrose in 9.9 mM HEPES pH = 7.4) with 10 U/mL papain (Worthington, Lakewood, NJ). After removal of papain, cells were triturated and plated at a density of 400–600 cell/mm^2^ on 12 mm coverslips (Thermo Fisher, Houston, TX) pre-coated with poly-D-lysine (Sigma, St. Louis, MO). Cultures were initially maintained in Basal Medium Eagle (BME) with fetal bovine serum, sodium pyruvate, and L-alanyl-L-glutamine (GlutaMAX; Invitrogen, Grand Island, NY) at 36°C and 5% CO_2_. After 2 hours, half the culture medium was replaced with Neurobasal Medium (Invitrogen) pre-conditioned for 24 hours over 1-3-week-old glial cell culture (conditioned medium), and then half of the conditioned medium was refreshed after 2 more hours. Cultures were treated with 1μM arabinoside-cytosine (Sigma) on day in vitro 3 (DIV3) to inhibit glial proliferation and refreshed twice a week with conditioned medium.

Corticotropin Releasing Hormone (CRH) (Bachem, King of Prussia, PA) was added to the medium on the 7^th^ day in vitro (DIV7) and refreshed on DIV10. Neurons were exposed for a total of one week to levels of CRH at 0.01, 0.1, 1, 10, or 100 nM. Cultured neurons were fixed using fresh 4% paraformaldehyde (PFA) on DIV14.

### Immunocytochemistry (ICC) for MAP2

Neurons were washed in 0.1 M phosphate buffer (PB) for 1 minute and then fixed with ice-cold 4% paraformaldehyde in 0.1M PB pH = 7.4 for 1 hour. They were then washed quickly in phosphate-buffered saline + Triton-X (PBS-T) and treated with a blocking buffer (3% NGS, 1% BSA, 0.3% Triton-X in 0.01 M PBS, pH = 7.4) for 30 minutes. Coverslips were treated with mouse anti-MAP2 antibody (Sigma) diluted 1:10,000 in 0.3% Triton-X in 0.01 M PBS at 4°C for 48 hours. Finally, they were washed 3 times in PBS-T (5 minutes/wash) and then incubated in AlexaFluor488 goat anti-mouse IgG conjugate at a concentration of 1:400 (Invitrogen, Grand Island, NY) in 0.3% PBS-T with 1% BSA at room temperature for 2 hours. After washing for 5 minutes in PBS-T 3 more times, neurons were processed for confocal imaging with aqueous mounting medium (Gel-Mount; Biomeda, Foster City, CA) to protect against photo-bleaching.

### Systematic imaging and analysis of dendritic length and arborization (Sholl analysis)

All coverslips were assessed and quantified without knowledge of treatment group. Each experiment included 12 coverslips per treatment group, and neurons were sampled equally from each coverslip for imaging. To objectively select neurons, each coverslip was imaged at lower resolution in four locations: a 1mm x 1mm square was 90° apart from each other and exactly 1 mm from the cover slip edge. These locations were selected in advance to obviate the potential confounder of high density of neurons and confluence of processes. All neurons with cell bodies within each 1mm x 1mm region and touching at least two neighboring neurons were imaged using confocal microscopy, Zeiss LSM 510 (Oberkochen, Germany). Images were taken using a 20x objective with 2x digital zoom. Overlapping images were stitched together using Fiji ImageJ (U.S. NIH, Bethesda, MD, USA) [38]. To limit the analyses to pyramidal-like neurons, somata were traced and fit to an ellipse, and the major and minor axes of the ellipse were measured. Neurons with a minor axis/major axis ratio of <0.5 were considered presumed interneurons and excluded from analysis. Dendrites of all remaining neurons that could be differentiated from the dendrites of neighboring neurons were manually traced and analyzed using the Simple Neurite Tracer plug-in [39]. Final n per group were 0 nM = 35; 0.1 nM = 24; 1.0 nM = 26, 10 nM = 30; 100 nM = 21. For total dendritic branching, two outliers in the 10 nM group (n = 30) were excluded using ROUT outlier method, with a false discovery rate of 5% [40]. No other group had outliers.

## Results

**Fig 2:**
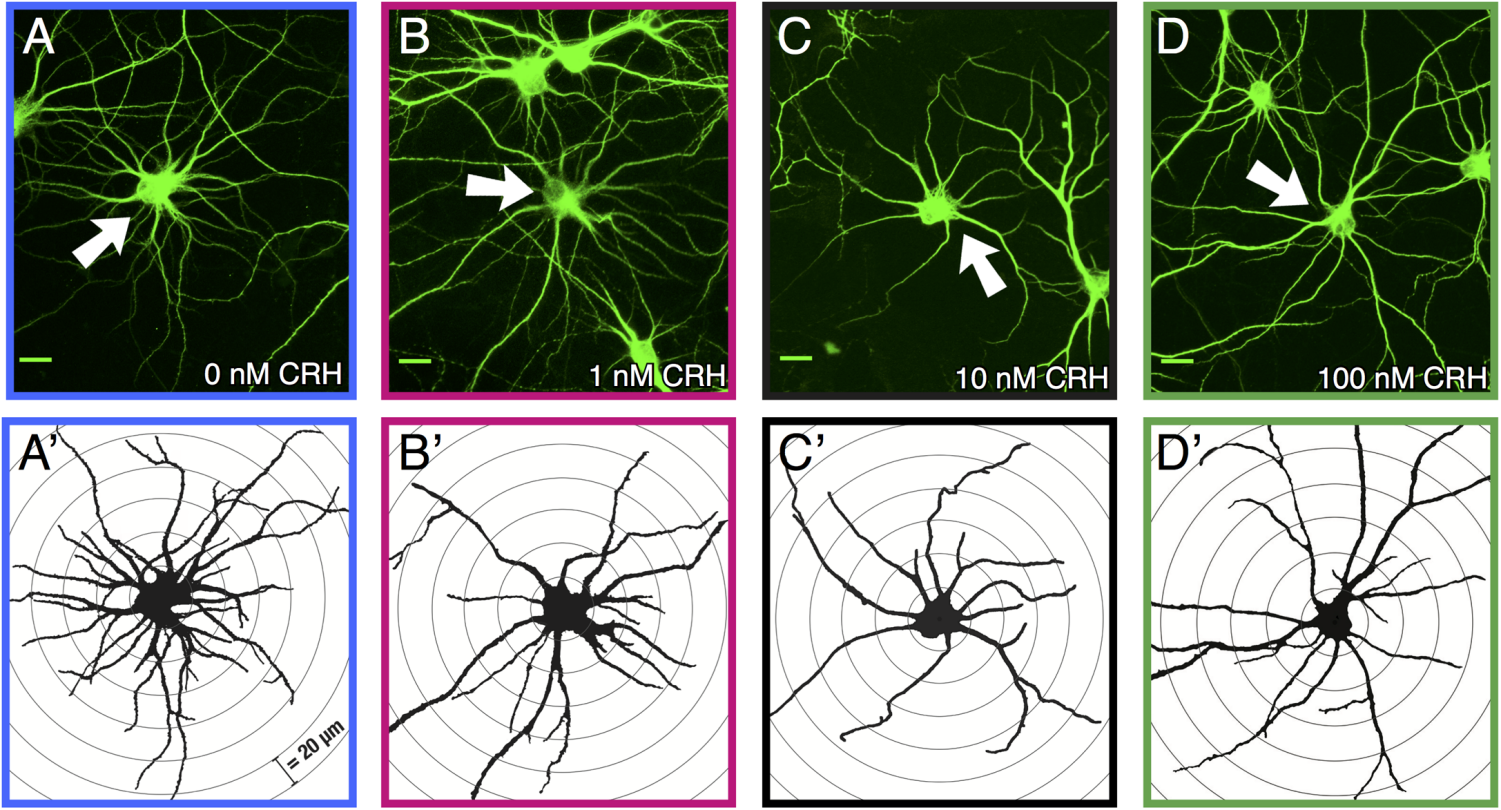
Images of cortical neurons treated with varying levels of CRH. A-D: 40x images of representative neurons exposed to 0, 1, 10, 100 nM CRH respectively. Scale bar is 20 μm. A’-D’: Traces of the same cells in A-D and concentric Sholl circles spaced 20 μm apart.

These experiments were designed to probe the plausibility of a direct causal role of CRH in mediating the effects of prenatal exposure to high levels of placental CRH on neuronal arborization and maturation that contributes to behavioral outcomes [29]. To this end, we used rat primary cortical cell culture, an *in vitro* model of neuronal development. Primary cell culture allows for the detailed analysis of neuronal dendrites from a neonatal rat, analogous, in terms of cortical development, to the 3^rd^ trimester in humans [41]. Neurons were exposed to chronic levels of CRH at 0.01, 0.1, 1, 10, and 100 nM for 7 days of treatment. Fig 2A–2D depicts representative neurons and Fig 2A’–2D’ are Sholl drawings of the same neurons (concentric circles at 20 μm distances). Sholl drawings are used measure dendritic branching, comparing the number of dendrites at varying distances from the soma.

**Fig 3:**
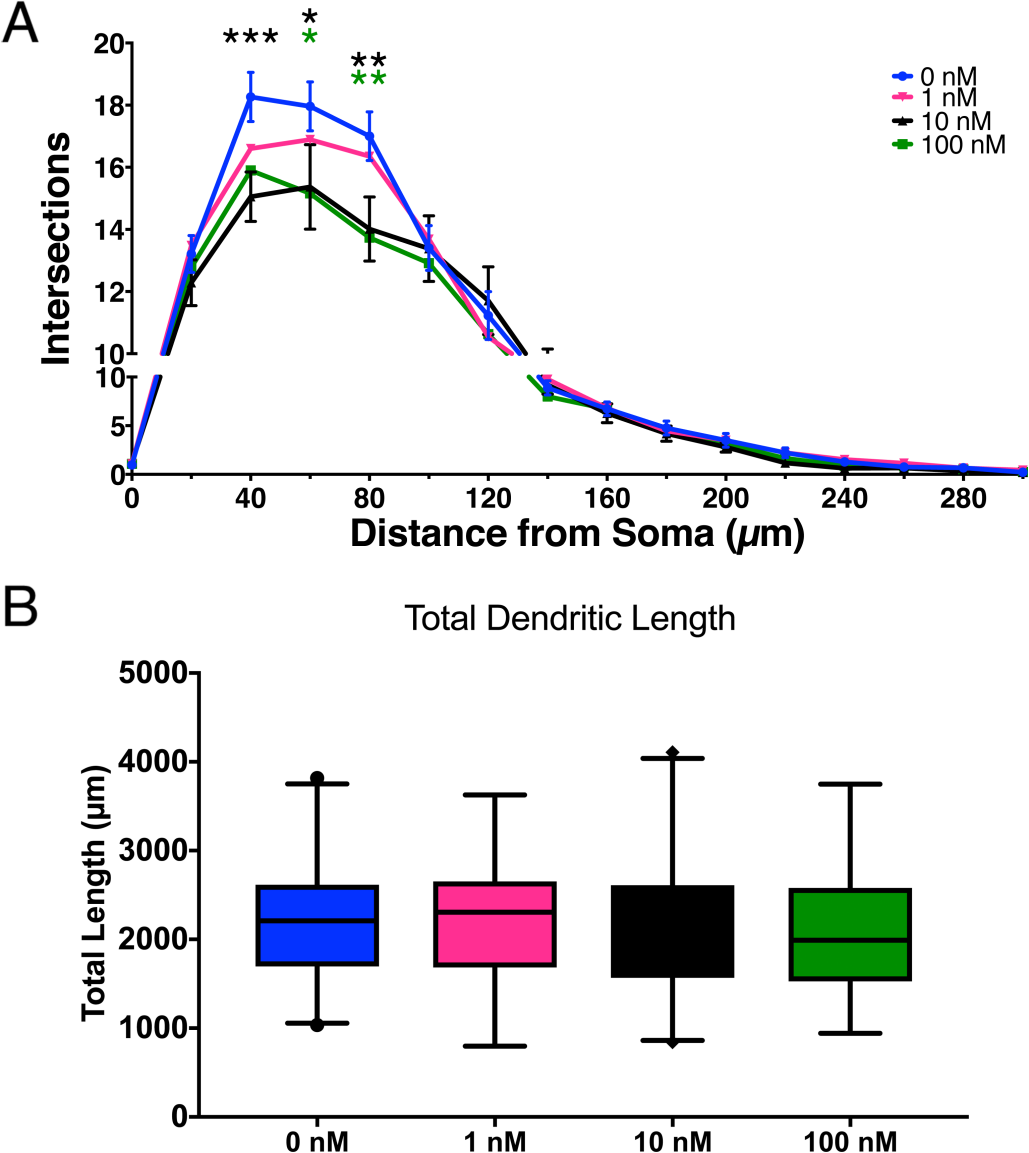
CRH exposure of developing cortical neurons influences dendritic branching, but not total dendritic length. A) Sholl analysis reveals the dose-dependent effects comparing varying levels of CRH. Significant decreases were found at 40 μm from the soma (10 nM, p = 0. 0006), 60 μm (10 nM, p = 0.009; 100 nM p = 0.014), and 80 μm (10 nM, p = 0.0017, 100 nM, p = 0025). Error bars represent SEM. B) Total combined dendritic length was not different between groups. (Error bars represent SEM. B) Total combined dendritic length was not different between groups Error bars represent 2.5–97.5 percentiles on box and whisker plots. For both figures, 0.01 nM and 0.1 nM had no significant effects and are not shown.

Chronic exposure to CRH led to a reduction in the dendritic branching of pyramidal-like neurons (Fig 3A). The effect was maximal at 40–80 μm from the soma (two-way ANOVA, Bonferroni correction for multiple comparisons, P<0.05). A dose effect was apparent at low concentrations but was not seen above 10 nM concentrations. Total dendritic branch length was also analyzed, but there were no significant differences found between treatment groups (Fig 3B) (one-way ANOVA, Bonferroni correction for multiple comparisons, p = 0.68). Dendritic branching is one of the components that drive dendritic length. Our data suggest that high levels of CRH likely inhibit the branching points of dendrites, probably by eliminating synaptic connections from other neurons, analogous to the known effects of the peptide [18]. Linear growth of dendrites and branches is not affected, and likely mitigates the overall effects on dendritic length

## Discussion

The principal findings of this paper are (1) cortical neurons at the developmental stage roughly concordant with late human gestation are sensitive to CRH; (2) CRH influences dendritic branching in a dose-dependent manner. Importantly, these effects are observed at CRH levels estimated to be present in the adult stressed brain [42].

Cortical pyramidal neurons are exposed to both local CRH, expressed in cortical interneurons [43] and potentially to CRH from other brain regions and circulating blood. Levels of CRH are low to undetectable in human blood, except during pregnancy when the human placenta produces large amounts of pCRH and releases it into both maternal and fetal circulation [44–46]. CRH produced by the placenta travels into the umbilical vein and enters fetal blood, and may thus reach the developing fetus [47]. Fetal plasma CRH levels throughout gestation are correlated with maternal plasma levels [48], stimulating the fetal pituitary to synthesize adrenocorticotropic hormone (ACTH) and drive fetal adrenal cortisol synthesis [47]. Once CRH enters the fetal circulation, it can also cross the blood brain barrier [33]. Thus, CRH has the potential to directly influence developing cortical neurons in the fetus.

Both the primate [49] and murine [12,13] cortex widely express CRH receptors, and those receptors can be found in fetal human neural stem/progenitor cells and in differentiated cortical neurons as early as 13 gestational week [32]. CRH at levels studied here has been found to directly influence the development of hippocampal neurons, including dendritic differentiation [18,50]. Although placental CRH is poised to influence cortical neurons as well, the direct effect of CRH on the structural development of cortical neurons had not been studied previously either *in vivo* or *in vitro*. The current study found that exposure to CRH directly reduces dendritic branching of pyramidal-like cortical neurons, indicating that the levels of this maternal stress hormone during fetal development likely mechanistically regulate the structure of the cortex.

In the current study, cortical neurons were exposed to chronic CRH at varying levels. There is little direct information about levels of CRH that bathe the developing human cortex. In the human cohort that our team has studied, we found maternal blood levels of placental CRH can reach as high as 3000 pg/mL (0.6 nM) [51], which approaches the levels that decreased dendritic branching. In our experiment, cortical neurons were only exposed to CRH for 7 days *in vitro*, while human fetal cortical neurons are exposed for months, allowing for more significant impacts. Additionally, CRH can cross even the developed blood brain barrier and may accumulate in fetal brain [33]. Finally, fetal cortical neurons are also exposed to endogenous CRH that is produced within fetal brain and has combined effects with placental CRH [52].

Here, the adverse actions of CRH on cortical neurons were only observed at higher levels tested. This is important, because all fetuses are exposed to pCRH as a normal part of gestation. The dose-dependent actions of CRH indicate that adverse cortical neuronal maturation will be limited to fetuses exposed to relatively high levels of pCRH.

Several negative effects of high levels of CRH on the developing brain have been demonstrated in rodents. The peptide increases neuronal excitability [22], promoting seizures and even neuronal death at high levels [23]. Whereas existing work has shown adverse actions of the peptide in hippocampus [22,50] and amygdala [22,53], this is the first report of untoward actions of CRH on the developing cortex. It is interesting to note that direct effects of CRH on the developing organism are found throughout evolution (1). For example, CRH is expressed in the Western spadefoot toad, which lays its eggs in pools of desert rainwater. If tadpoles detect that the conditions for normal development are unfavorable (e.g. rapid evaporation of the pool), CRH accumulates in the hypothalamus, promoting metamorphosis and escape from imminent peril. Notably, here, too there are adverse effects of high levels of CRH: tadpoles that survive by accelerating their development are smaller than average at emergence as toads and have reduced capacity to forage for food and to mate (2,3).

Placental CRH production increases in response to maternal signals of stress which is associated with maternal glucocorticoid (cortisol) production. In the hypothalamus, glucocorticoids inhibit CRH transcription providing a negative feedback during stress. Surprisingly, in the placenta, cortisol promotes production and release of pCRH [54], constituting a ‘positive feedback’ system. Thus, relatively high levels of cortisol and of CRH may both be present in the stressed pregnant mother [25,55], and steroids and pCRH can act synergistically to change neuronal structure [56]. The current work reveals for the first time that CRH alone suffices to alter neuronal development by stunting dendritic branching. Namely, the peptide is not merely a marker of stress or other conditions influencing fetal neuronal maturation via CRH-independent mechanisms. These effects of the peptide are important, because neuronal dendritic arbors form the bulk of cortical volume [31,57]; thus, changes in dendrites are likely to be reflected as measurable changes in cortical structure and function. Taken together, these data indicate that direct actions of CRH on cortical neuron maturation may provide a novel mechanism by which prenatal maternal signals have a lifelong effect on the neuropsychiatric outcome of her child.

## Supporting information

S1 File This includes all original data for the dendritic length and Sholl analysis that produced the results found in the study. (XLSX)
